# Supplementary figures and images for: Integrating across neuroimaging modalities boosts prediction accuracy of cognitive ability
Source: PLoS Comput Biol. 2021 Mar 5;17(3):e1008347. doi: 10.1371/journal.pcbi.1008347 (PMC7984650; doi:10.1371/journal.pcbi.1008347)

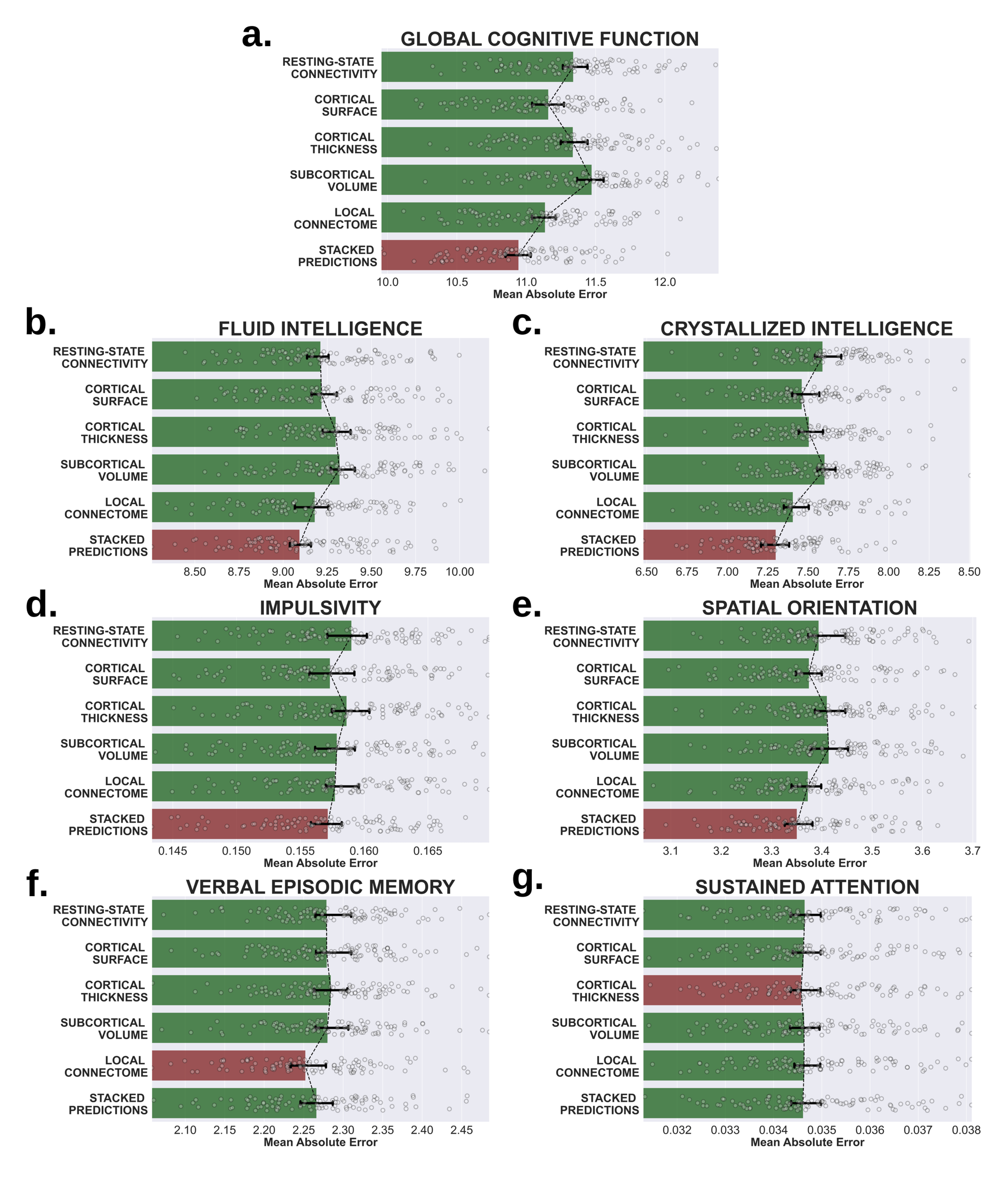

Supplement: S1 Fig — Mean absolute errors (MAE) between the observed and predicted values of seven cognitive scores using each brain measurements separately and together by stacking their predictions. In red the scenario that yields the maximum score. (TIF) [file pcbi.1008347.s001.tif]

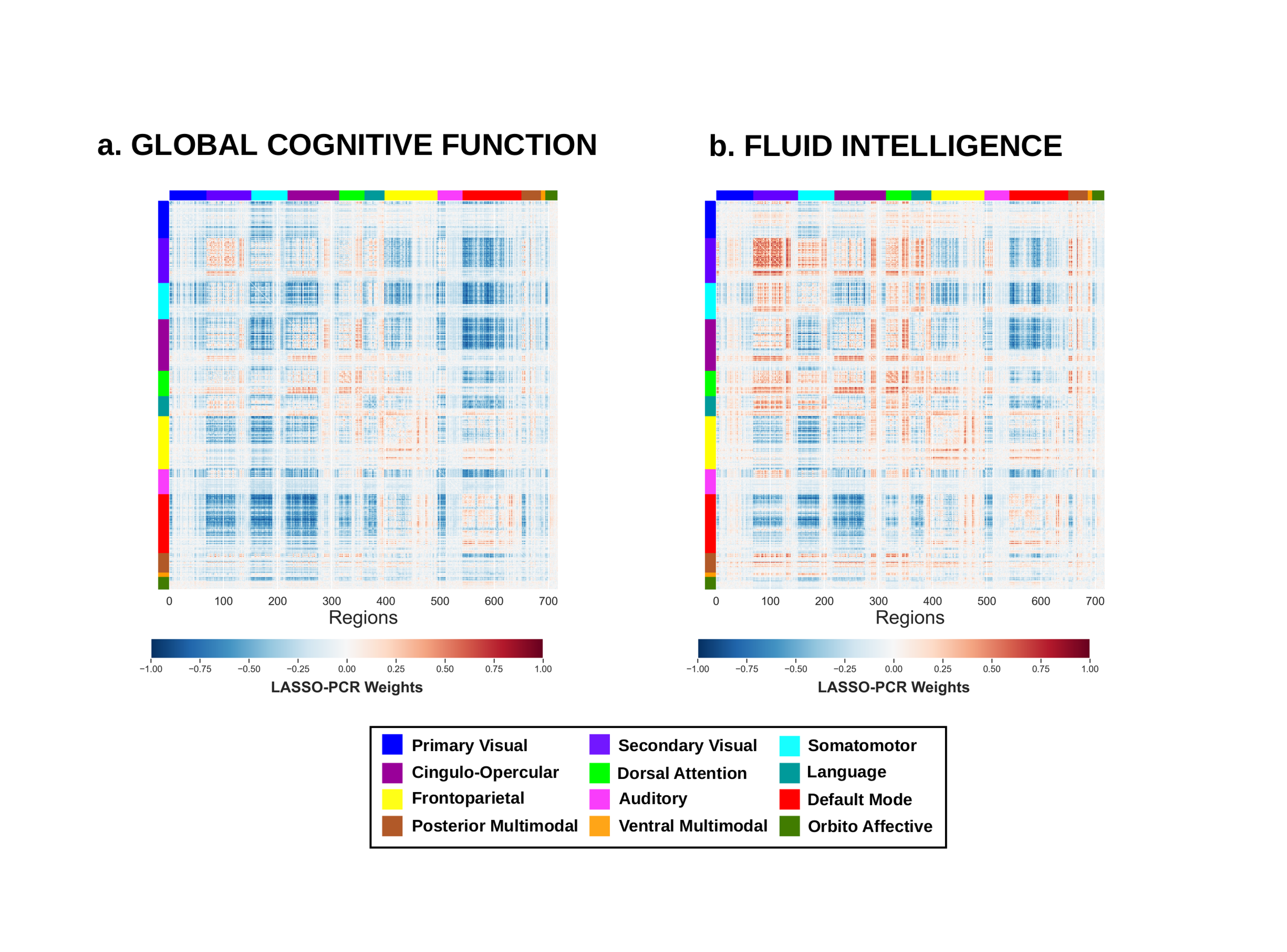

Supplement: S2 Fig — Encoding weight maps from the resting-state connectivity matrix coefficients for the two cognitive areas (global cognitive function and fluid intelligence) in which these attributes significantly contributed to stacking. Red and blue colors display positive and negative weights respectively. Loadings have been scaled to a [-1, 1] range without breaking the sparsity using the function maxabs_scale in scikit-learn. (TIF) [file pcbi.1008347.s002.tif]

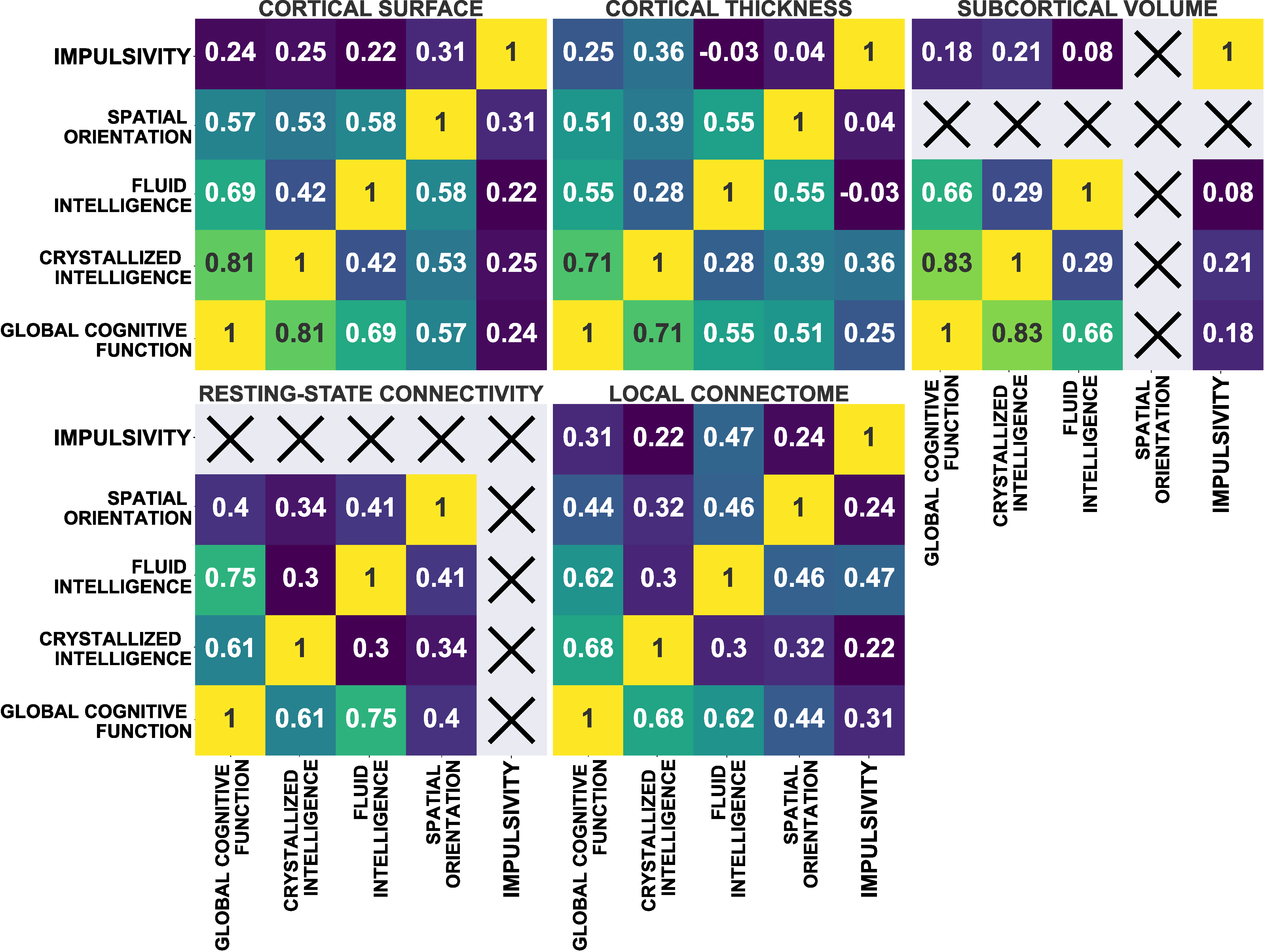

Supplement: S3 Fig — For each brain measurement, the Pearson correlation coefficient is computed to assess the similarity between the brain correlates of each cognitive score in which stacking led to a significant performance enhancement. A cross along domains for a given brain measurement indicates that the LASSO-PCR model failed to keep any feature during the optimization process. (TIF) [file pcbi.1008347.s003.tif]

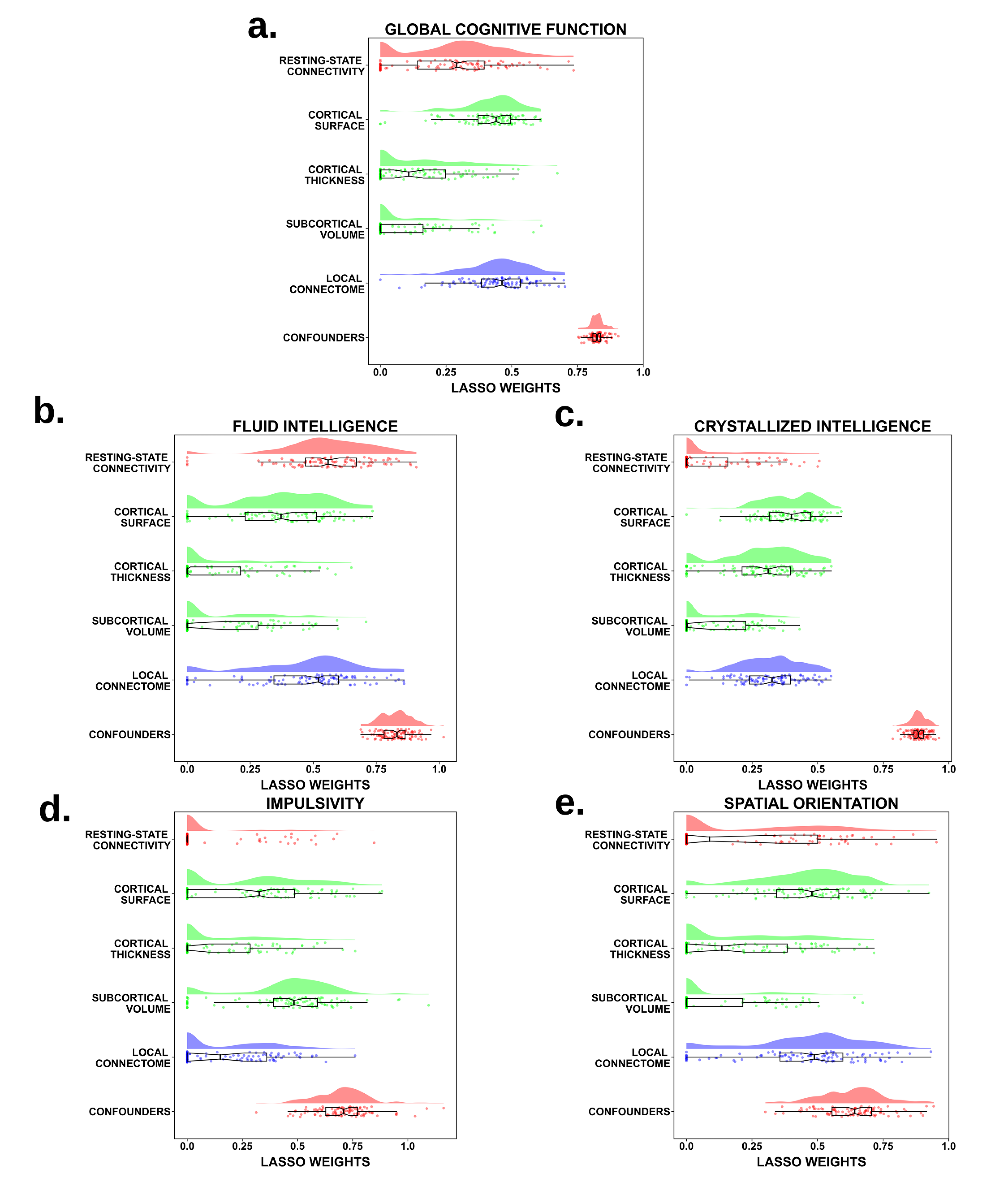

Supplement: S4 Fig — Across the 100 different data splits, the weight distribution assigned to the out-of-sample predictions of each brain measurement by the stacked LASSO model that includes also a channel for confounders (gender, age and education level) in those original cognitive scores in which stacking significantly improved the overall performance. (TIF) [file pcbi.1008347.s004.tif]

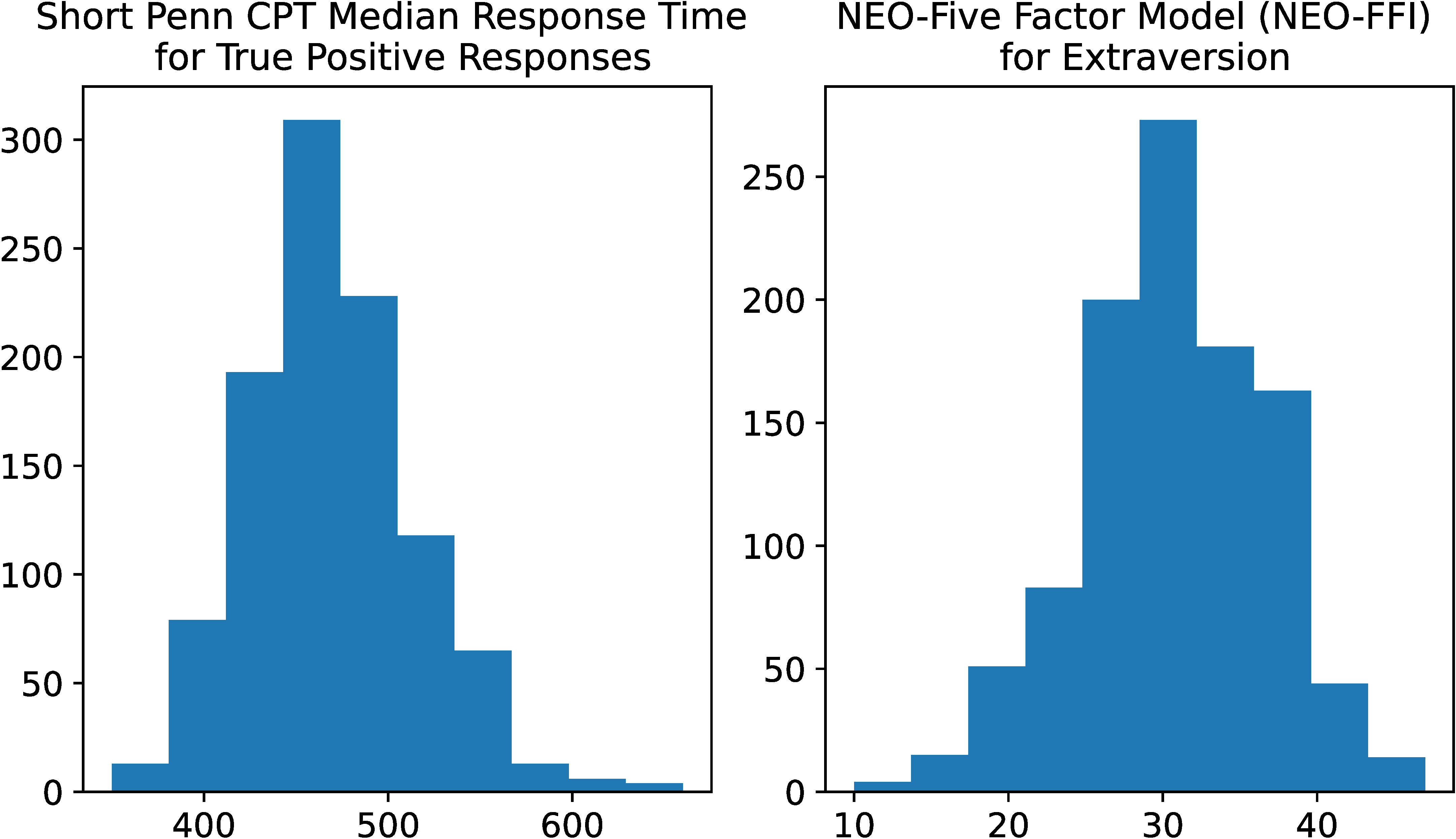

Supplement: S5 Fig — Univariate distributions for the Five Factor Model (NEO-FFI) score for extraversion, a proxy for impulsivity, and the Short Penn CPT median Response Time For True Positive Responses for sustained attention. (TIF) [file pcbi.1008347.s005.tif]

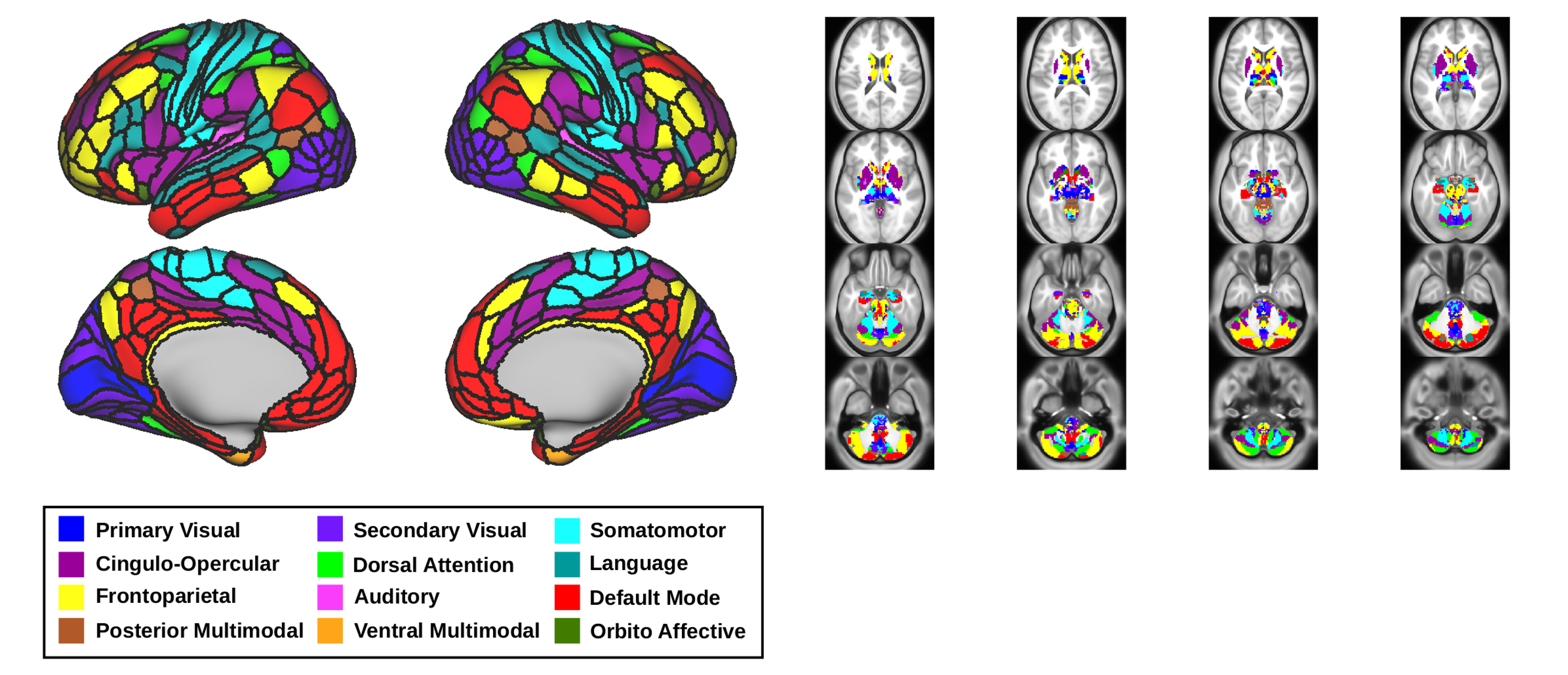

Supplement: S6 Fig — A parcellation consisting of 718 regions, for which 360 are cortical regions and the remaining subcortical. In colors, their assignment to 12 major resting-state networks as provided in [54]. (TIF) [file pcbi.1008347.s006.tif]

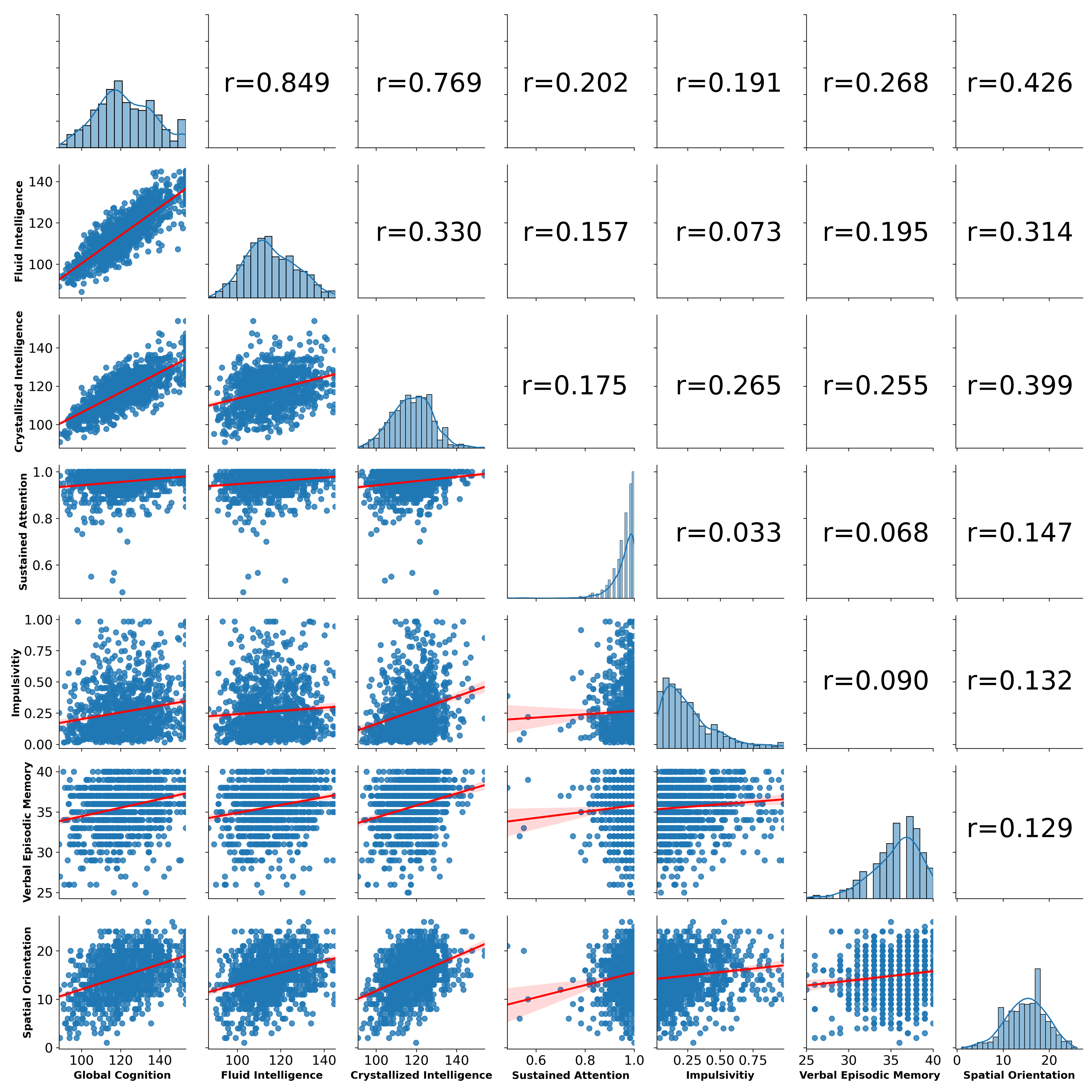

Supplement: S7 Fig — A pairplot where the diagonal shows the univariate distributions for the response variables used in our study and the off-diagonals the similarity between them, visualized by means of scatterplots and quantified using Pearson correlation coefficients. (TIFF) [file pcbi.1008347.s007.tiff]
