# Supplementary material for: Integrating across neuroimaging modalities boosts prediction accuracy of cognitive ability
Source: PLoS Comput Biol. 2021 Mar 5;17(3):e1008347. doi: 10.1371/journal.pcbi.1008347 (PMC7984650; doi:10.1371/journal.pcbi.1008347)
Supplement: Attachment — Submitted filename: R1_response_letter.pdf [file pcbi.1008347.s011.pdf]

We would like to thank the reviewers for their time and thoughtful critiques, which were highly constructive and led to beneficial revisions that help to raise significantly the quality of the paper.

Before going into detail describing the revisions to each point raised by the reviewers, we would like to mention the major changes included in this revision version:

- We have adjusted for several confounders, which included the intracranial volume, gender, age and education level.
- We have adjusted the cross-validation procedure to take into account the twins' information presented in the Human Connectome Project.
- We obtained patterns of feature importance that have been transformed in order to take into account important caveats that directly impact interpreting the weights in backwards or decoding models.
- Now the second level LASSO model restricts to the estimation of nonnegative weights. This has been proven to be beneficial when using stacked regressions (Breiman 1996) and more importantly, helps the interpretation of the contributing channels to stacking.
- We have changed the layout in Figure 4 for the weight maps, which we believe helps the comparison across modalities and brain measurements.
- We have changed the Figure 1 to a more detailed schematic version, which we believe is less confusing and more informative than the previous one.
- We have tried other response variables where the distribution of our original cognitive scores could affect the predicted performance in order to show the robustness of our findings in these cognitive domains.
- We have added several sub-sections to the Materials & Methods section for further clarification.
- Results have been updated accordingly to the new analyses performed.
- We have added new supplementary figures related to the new analyses performed.
- We have also included a supplementary figure showing the distributions and similarities between the response variable used in our study.
- We have included a supplementary figure showing the parcellation used in our study.

References:

- Breiman L. Stacked regressions. Machine Learning. 1996;24(1):49--64.10.1007/BF00117832

In our replies below, the reviewer comments are in black and our replies are in blue.

Reviewer #1

Reviewer #1: In this paper, the authors investigate prediction performance for behavioral measures in a large open dataset of young healthy adults when using several neuroimaging modalities with a transmodal learning approach. Overall the topic is of interest for the community and the paper is well-written, the main conceptual ideas are well-communicated for a large readership. I nevertheless have methodological concerns that should be addressed in order for the paper to be further considered for publication.
Main first main concern is related to the interpretation of the features contributing to the prediction based on the weight in the LASSO. This type of postdoc interpretation has been shown to be dangerously misleading. Clear demonstration, discussion and recommendations are provided in Haufe et al. (2014).

Reply: We agree with the reviewer about our misleading interpretation of the weights from the predictive LASSO-PCR models to each single-channel. We are deeply grateful for bringing this manuscript to our attention as it will affect for the better our future work in this area. Therefore, following the recommendations provided in Haufe et al (2014), we have transformed our predictions patterns, *i.e.*, those weight maps in the prediction models, to encoding weight maps by multiplying them by the data sample covariance matrix of each single-channel, thus permitting a direct interpretation of the weights. As such, throughout the manuscript these encoding weight maps are what we report and interpret. We have also uploaded the original decoding weight maps to *figshare,* in case anyone is interested in using them. These could be seen as deployed fitted models for a further use on external independent datasets.

In addition, we have added a new sub-section called "Weight maps of feature relevance" to the Material & Methods in which we talk about all of this with detail.

My second main comment pertains to confounding factors. It seems that the authors haven't carefully controlled for other factors than age, in particular they did not control for the effect of brain size (with TIV or ICV) which often spuriously influence covariance between neuroimaging features and behavioral features (partly through the effect of gender).

Reply: We share the reviewer's concern and we regret that we did not consider this at first. Thus, we have re-examined the adjustment for possible confounders in our analyses. In particular, we have considered two possible sources of confounders: neuroimaging confounders, represented by the intracranial volume ICV, that directly affect the properties and brain, and mediating confounders, represented here by gender, age and education level, that indirectly affect the link between the brain properties and cognition. Such a distinction led us to consider a different adjustment strategy for them. For ICV, we have regressed out its effect from the response variable, using again the estimated coefficients only from the training dataset in order to avoid any data leakage. For the mediating

confounders (age, gender and education level), motivated by a recent work by Dinga et al (2020) in which it is recommended to control for confounders at the machine learning predictions level, we took advantage of our stacking learning scenario to include this group of variables as an additional single-channel. If these confounders were to carry all the variability of cognitive prediction, then our second level LASSO model would just pick the predictions from this channel. Essentially this evaluates the "value added" by the neuroimaging terms to predicting cognitive ability above and beyond major social predictors. As we now show in the new Results sub-section "Prediction adjustment for non-neuroimaging confounders", even with the addition of this confounder channel, the neuroimaging channels were still contributing to the stacked model in a similar relative way.

In addition, we have added a new subsection to Material & Methods called "Confounding adjustment", in which we describe the details of this hybrid strategy for confounders control.

Reference:

- Richard Dinga, Lianne Schmaal, Brenda W.J.H. Penninx, Dick J. Veltman, Andre F. Marquand bioRxiv 2020.08.17.255034; doi https://doi.org/10.1101/2020.08.17.255034

My third main comment is related to the selection of the behavioral scores of interest. The choice was not supported by any arguments. It is good that the authors tried to cover different behavioral domains, however, many of the scores appears actually unpredictable based on brain data. The distribution of the unpredictable behavioral measures could be questioned. The authors gave some specific summary measures but could they provide visual illustrations of distribution (basically histograms) while taking care of showing the whole range of possible values in each test on the x axis?

Reply: Excellent point. As we acknowledged in the Discussion section, the properties of the distributions of the response variables could be a possible source of poor performance when trying to make predictions in certain cognitive domains. In order to supply more information about the distributions of our response variables, beyond what is reported in Table 1, we have added to the Supplementary Material a pairplot like the one presented below. This figure is important for two reasons. First, one can clearly visualize the distribution of each dependent variable. Second, it assesses and quantifies the similarity between these scores, which give a further justification of the redundancy that we observe in our weight maps across some cognitive domains.

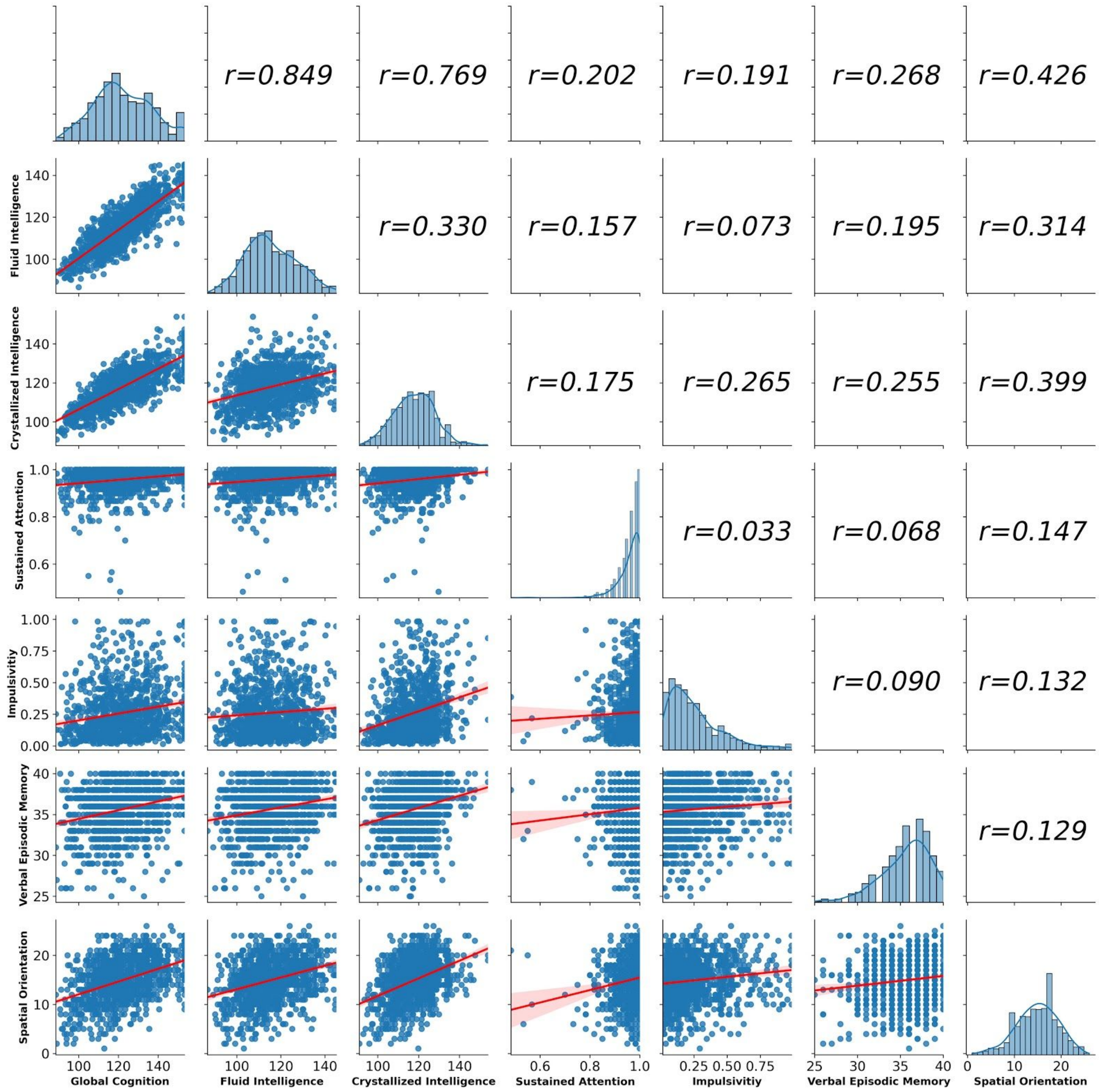

I suspect that some scores have ceiling effect and lack relevant interindividual variability that would be needed to get any predictive power as partly discussed by the authors on line 422. Some scores could be replaced by scores for which relatively decent prediction performance have been reported in other studies. For example, impulsivity could be replaced by extraversion.

Reply: This suggestion allowed us to explore, in more detail, the effects of the response data distributions on the predicted accuracies. As a consequence, and following the reviewer's recommendation, we have repeated our analysis for impulsivity using the Five Factor Model (NEO-FFI) for extraversion, and the Short Penn CPT median Response Time For True Positive Responses for sustained attention. Both of these have distributions that are more Gaussian than the original cognitive variables for these same constructs.

As shown below, and compared to our original scores in these cognitive domains, the distributions for these new variables look more “friendly” (i.e., more aligned to the assumptions of the distributions of the residuals for a sparsity constrained GLM model). Interestingly, when predicting on them, we found that both single-channels and the stacked model predict worse than random guess (R^2<0). If the reviewer is interested, these results can be found at the end of the notebook called “05-predictions.ipynb” in the Github repository for the project that is linked in the main text. This supports that, at least for these cognitive domains, the distribution properties of the scores used did not play a big role in predicting these cognitive domains.

We have thus added a new small sub-section called “Testing for ceiling and floor effects” to the Results section, which reads:

“In order to rule out the possibility that ceiling and floor effects of some of the scores might be influencing the poor performances obtained, we repeated the analyses for sustained attention and impulsivity using respectively the Short Penn CPT Median Response Time for True Positive Responses score and the NEO-Five Factor Model (NEO-FFI) score for extraversion, which both appeared to exhibit more friendly distributions (see Fig S5). Interestingly, for both scores all single-source channels as well as stacked predictions performed worse than random guess ($R^2 < 0$), supporting the effect sizes observed in these domains for the original scores.”

We have also added a line to the Discussion section, connecting these results to the part in which we talk about the distribution properties of our response variable as a possible limiting effect in our predictions:

“..However, as we also showed, even after replacing these scores for others with more desirable distribution properties, prediction accuracies did not improve, which highlights how challenging it can be to predict individual differences of cognitive performance using neuroimaging data.”

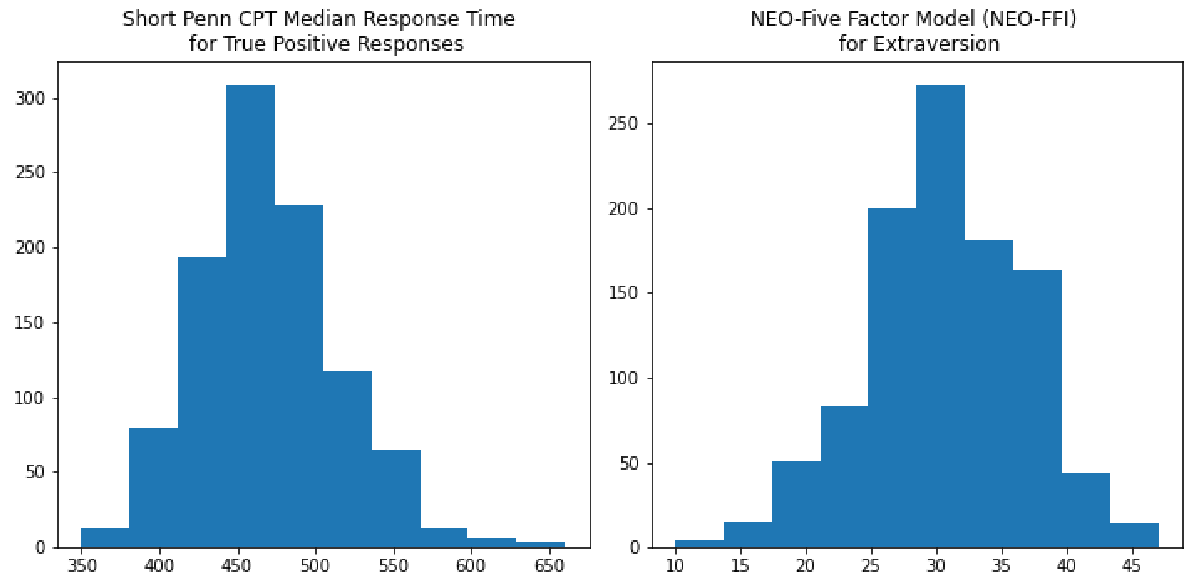

Furthermore, it was not clear whether the family structure was taken into consideration in the cross-validation scheme? having family members split (in particular twins) across folds could result in overoptimistic performance (the model trains on a group of subjects would work better on the relatives of those subjects than in unrelated individuals).

Reply: Our dataset included 419 observations with twin zygosity information as verified by genotyping. We obtained this odd number due to some of the twins being discarded during the data preparation process, either due to the lack of a measure in any of the response variables used or the lack of reconstructed intracranial volume information.

Thus, we generated our 100 cross-validation partitions ensuring that twins either fell in the training set or the test set and never in both at the same time, so that no relative information can be exploited to inflate the results.

We have created a new sub-section called “Cross-validation strategy for performance assessment” to the Materials & Methods section where, among other things, we comment on how we handled these related twin individuals.

Minor comments:
Introduction:
Introduction should be more balanced: machine learning approaches suffers from a lack of interpretability and despite all the promises and perspectives that have been suggested by several review pieces, the contribution of these approaches to our understanding of brain-behavior relationships remains currently very limited, if not null. Issues related to false positive, replicability, and sample size are illustrated and discussed along the same lines in Kharabian Masouleh, Eickhoff, Hoffstaedter, Genon, and Alzheimer's Disease Neuroimaging (2019).

Reply: We agree with the reviewer that the introduction lacks a description of the limitations of machine learning multivariate approaches, particularly when compared to univariate

analyses. Therefore, following this suggestion and the reference provided, we have added the following lines to the end of the second paragraph in the Introduction section:

"...Nevertheless, these multivariate methods do suffer from problems with interpretability. For example, in the pursuit of maximizing performance, some approaches may rely on complex non-linear models (e.g., deep neural networks), for which directly assessing feature importance can be challenging. Even in relatively simple multivariate linear models, which establish a transparent relation between the input features and the response variables under investigation (e.g. sensory, cognitive or task conditions), large weights that carry no signal-of-interest whatsoever can emerge [16]. In this regard, univariate methods are more straightforward to interpret and therefore, it has been argued that multivariate and univariate analyses should be considered complementary when exploring brain-behavior associations [17]."

What is the meaning of "A multi-modal cognitive phenotype"? A cognitive phenotype refers to the expression of a specific cognitive pattern, for example, low episodic memory performance together with low spatial navigation performance and low extraversion score in a group of individuals. Here, the authors are actually referring to a multi-modal brain phenotype that related to cognition.

Reply: We agree with the referee that the term "A multi-modal cognitive phenotype" is poorly formed to describe the process we are investigating. As such, we have dropped the term "cognitive" from the name. Moreover, we have cut the term "phenotype" from the text and replaced it by either weight map or weight pattern (both are interchangable) to be more specific.

Results:
Line 158: "with the former contributing significantly more than the latter", what is the statistical test here ?

Reply: We have focused on whether intervals overlapped or not. If they do not, we can safely say that they are significantly different. When they overlapped and we wanted to make any statement of their significant differences, we used an appropriate statistical test (as for example we do in line 157 in the clean Manuscript, that is, without the tracked changes).

"Such diverse patterns of contributions from single-channels to the stacked model may be partially affected by the L1 regularization term dealing with the shared variance between predictions" this actually shows that the interpretation of the weights following LASSO should be avoid.

Reply: Indeed. We were not aware of this and we are really grateful to the reviewer for bringing this issue to our attention.

Line 179, "anterior parietal areas" is relatively fuzzy, to which anatomical regions do the authors actually refer to?

Reply: We agree that such a term was fuzzy. As a consequence, we have better specified the regions we were referring to.

Overall, cortical thickness and cortical surface weights go against what could be expected from the neuroscience literature: positive associations with high-level cognitive scores are found in regions around the central sulcus and even in visual cortex for fluid intelligence, while negative associations are found in most of the association areas. A similar concern can be raised for association of global cognitive ability with brainstem pathway. How did the authors explain these puzzling findings ?

reply: Thank you for bringing this to our attention. We agree that some of the findings for cortical thickness and cortical surface areas might be counter-intuitive. Some of these patterns have changed with the revised way of presenting the weight maps (as per the comment above). Interpreting multivariate weight maps are generally a bit more complicated and nuanced than interpreting univariate maps (e.g., voxelwise GLMs). Correlations between areas can impact whether a region positively or negatively loads in the prediction of a particular response variable. What this means for interpretations is that, as a whole, an increase in the value of certain regions will lead to an increase (or decrease) of the response variable when other regions are themselves increasing or decreasing. This sometimes leads to counterintuitive interpretations of regional encoding.

In order to further support our results regarding the weight maps from cortical thickness and surface area properties, we have used the exact Freesurfer information which can be found in the unrestricted data provided by the Human Connectome Project. This information corresponds to the cortical thicknesses and surface areas for the Desikan atlas (68 cortical regions). If our findings are correct, we should observe a similar pattern with slight variations due to using a different parcellation.

These weight maps, for predicting global cognitive scores, are shown in the figure below. As we can see, the obtained patterns, using an already provided input data without any manipulation, are qualitatively similar to those obtained in our manuscript using a different parcellation. We believe this supports our findings.

We kindly invite the reviewer to check these wight maps computation at the end of the notebook “08-analyse_and_plot_phenotypes.ipynb” in the repo included with the manuscript

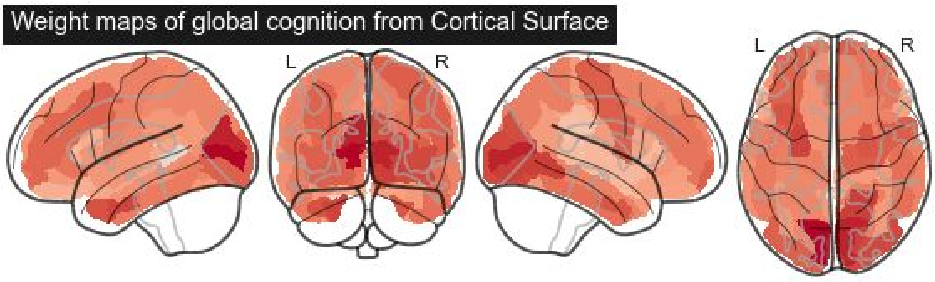

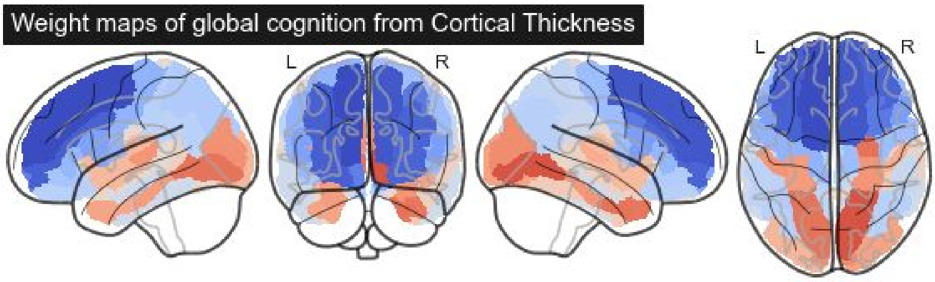

Discussion:

Line 378: “mean global BOLD signal, which is supposed to strengthen…” this sentence is confusing, currently it can read as global signal strengthen the association while this is the global signal regression procedure that strengthens the predictive power.

Reply: We agree with the referee that this sentence is confusing. We have changed the whole sentence, which now reads as follows

“...In addition, it is important to note that our preprocessing pipeline does not include a global signal regression step, which is supposed to improve resting-state functional connectivity based behavioral prediction accuracies…”

Reference:
Haufe, S., Meinecke, F., Görgen, K., Dähne, S., Haynes, J.-D., Blankertz, B., & Bießmann, F. (2014). On the interpretation of weight vectors of linear models in multivariate neuroimaging. Neuroimage, 87, 96-110.
Kharabian Masouleh, S., Eickhoff, S. B., Hoffstaedter, F., Genon, S., & Alzheimer's Disease Neuroimaging, I. (2019). Empirical examination of the replicability of associations between brain structure and psychological variables. Elife, 8. doi:10.7554/eLife.43464

Reviewer #2:

Reviewer #2: Integrating across neuroimaging modalities boosts prediction accuracy of cognitive ability
Javier Rasero, Amy Isabella Sentis, Fang-Cheng Yeh, Timothy Verstynen

Summary
This study tests whether the prediction accuracy of cognition can be improved by combining multiple imaging modalities. Using data from the human connectome project, the study combines features from diffusion, resting state, and structural MRI in a stacked model. Separate Lasso regularized regression models are first fit for each modality, and then the predictions from all modalities are used as input features into a second level lasso regression. The results show improvements of the combined model over and above each individual modality prediction.

General
This paper tackles a worthwhile question in a straightforward way. It is nice to see the authors avoiding overly complex models, and the article is written very clearly and reproducibly. I only have a few fairly minor comments. Please find my detailed comments below, approximately in order of importance.

1. The individual modality models are first optimized in cross-validation loops, and then the cross-validation of the second level model is performed separately. I wonder if it would be better to optimize both the first regression models and the second level model within the same cross-validation loop. Analogous to figure 1 in Dinga et al (https://doi.org/10.1016/j.nicl.2019.101796), we could think of the modality-specific models as the feature selection for the second level model. I would be interested in the authors' thoughts on all-in-one cross-validation versus the separate cross-validation loops that are currently in the manuscript.

Reply: We thank the reviewer for bringing this up. We'd like to emphasize that the outer cross-validation, *i.e.*, the way in which observations are splitted into training and test sets, is the same for both levels of learning. It is true that it would have also been possible to accommodate the same inner cross-validation, used in both cases for optimization. In any case, the importance here is just to keep the outer cross-validation the same across learning levels in order to stack predictions across the same observations in both stages. We believe that using the same inner cross-validation for optimization would just carry minimal changes to the predicted performances.

2. The figures of the resting state weights in figure 4 are not particularly informative. It seems that each of the networks has similarly strong negative and positive contributions. Without knowing the spatial distribution of these, it is hard to interpret these results. Perhaps

something like figure 2 in Smith et al (https://doi.org/10.1038/nn.) would be more informative?

Reply: We completely agree with the reviewer that subplots in figure 4, particularly those for the resting-state connectivity channel, had little informative value. In fact, deciding the best way to informatively report these weights from a matrix of 718 x 718 was one of our main visualization concerns. We appreciate the reference provided as it has given us very good ideas. Following these, and regarding again resting-state connectivity features, we have decided to plot the strength maps as the sum over rows (or columns) in the 718 x 718 weights matrix, taking the absolute entries and concentrating only on the 1% largest absolute weights. We have decided to use the absolute values and forget about the weight directions because, in contrast to the rest of modalities, pearson correlations carry themselves a different consideration in terms of correlation and anticorrelation. To avoid possible misinterpretations, we resort to analyzing the size of the weights. We believe that this will provide a general flavour about the spatial pattern of importance using this channel. In any case, we still give the full matrices of weights in the figure S2, so that anyone interested in further exploration can be advised to examine these. We also share these matrices in the public Github repository for the paper.

In addition, as noted at the beginning of this letter, we have modified the layout in which we show the weight maps across modalities and cognitive domains in figure 4. This improves the general interpretability of the weight maps for all modalities, including the resting state weights.

3. In the participants section, I suspect that some of the details about the human connectome project are incorrect. There are two HCP's, and I think the PIs described here are not for the HCP data that was used here. For the young adult HCP (https://www.humanconnectome.), the PIs are David Van Essen and Kamil Ugurbil. Also, the universities that participated in the collaboration reflect the wrong HCP project.

Reply: We thank the reviewer for pointing this out to us. We have indeed mixed information about both studies. Thus, we have accordingly changed this information in the participants sub-section to just WU-MInn HCP, which is the study we are using. In addition, we have also changed the acknowledgments sentence according to https://www.humanconnectome.org/study/hcp-young-adult/document/hcp-citations

Reviewer #3

Reviewer #3: This paper provides evidence that combining different MRI modalities accounts for a slightly larger portion of variance in cognitive abilities and attributes than each of modality alone. To test this idea, the authors analyzed a large dataset from the Human Connectome Project, which includes multi-modal MRI measurements and measurements of a battery of cognitive and behavioral assessments. The results demonstrate the combining features from different modalities through a stacked twice-regularized linear regression model can provide a small (1-4%) but consistent boost in variance explained, compared to the best uni-modal regularized regression model. These results are important because, while projects such as HCP are collecting multi-modal data, it is still rather unusual that data are combined across modalities to account for behavioral variance. This result motivates further exploration of these relationships, and shows the way towards a principled approach to do this, with structured/interpretable machine learning approaches.

Overall, the paper is well written, the analysis methods are of high quality and the interpretation of the results is sound. The high level of technical proficiency and transparency in sharing of code and data are particularly remarkable, and provide clarity about the methods and results well beyond the current standards of the field. The use of non-parametric permutation statistics for analysis of statistical significance is also a strength. That said, I think that the some of the choices made in setting up the comparisons are not clear or not sufficiently well-motivated. Testing and comparing alternative approaches to this analysis may provide more direct and possibly also more powerful evidence of the original hypothesis.

My main concern is that, beyond reference to previous work that used such an approach (Liem et al., 2017 and the original Wolpert work), it is not clear why the stacking architecture is chosen here, and whether this choice is beneficial. In particular, among the examples cited, this is the first to use linear models with the stacking architecture, and it is not clear that this combination makes the most of the integration across modalities. For example, one hypothesis about the relationship between brain features and complex cognitive constructs is that many small and distributed biological effects all add to explain the variance in cognition (see e.g., https://www.biorxiv.org/content/10.1101/2020.09.01.276451v1 and references therein). It seems that the stacking architecture is designed to specifically suppress these kinds of cumulative effects, particularly across modalities.

An alternative to the stacking model would have been to compare the modality-specific models to a LASSO-regularized model that includes all of the features across all modalities. It is possible that this would have (1) provided even higher variance explained and (2) provided more information about redundancy and complementarity between the models. For example, some of the data from different modalities pertains to similar anatomical locations (e.g., cortical thickness and resting-state connectivity of the same parts of cortex). The current model architecture doesn't allow the model to aggregate these across modalities, and improve the estimation SNR through this aggregation. Meanwhile, a LASSO over all of the features would help adjudicate whether different features extracted from the same

anatomical location are redundant, and whether one is reliably selected over the other, or whether they combine to provide higher accuracy. This is because the stacked architecture strictly limits the interactions that can take place between the features at the first level, only allowing features that are important at the unimodal level to come through.

Reply: We are grateful to the reviewer for these thoughtful comments on our approach. Indeed, if we understand the comment correctly, the referee is referring to something like group LASSO (or a PCR/Group LASSO variant). While we had considered this idea, these sorts of all encompassing first level models have several drawbacks. First, methods like group LASSO still assume that the characteristics of the noise across groups will have similar distributional properties. This is not the case with the different imaging modalities (and now demographic modalities) that were tested here. Second, a critical advantage of stacking is the dimensionality compression: it reduces high dimensional problems into a single dimension (i.e., the prediction) and evaluates across feature sets (i.e., channels) independent of dimensionality. This presents a unique advantage when working with the extremely high dimensionality of neuroimaging data. Collapsing across all channels in a group LASSO approach would still ultimately fail to handle the dimensionality of these data sets.

We acknowledge that this approach is not the only one that could have been taken and that can exhibit some limitations, particularly about the lack of interactions between features from different modalities that could improve overall performance. Regardless of this, we consider our scenario a first approximation open to further future improvements. In any case, it is also important to emphasize the strong points of about scenario, for which we have then added the following paragraph to the discussion section:

"On the other hand, it is worthwhile emphasizing the justification of our stacking learning approach compared to, for example, a simple regression model that includes all of the features across all modalities. First, our framework effectively estimates the unique variance explained by each neuroimaging modality in predicting cognitive performance, acting thus as a form of feature selection at the neuroimaging modality level that accounts for redundancies in correlated signals. Second, the distinct underlying noise structure across modalities is handled in our approach by fitting each single-channel independently, in contrast to linear regression models using all the data together. Finally, our predictive framework shares some characteristics with resample-based ensemble methods (e.g. random forest), which in some cases might be a more efficient way of handling wide datasets (number of features exceeding the number observations). For example, in a supplementary analysis predicting global cognitive function (not shown in Results), we found that the same LASSO-PCR procedure applied to the concatenated data across modalities (a matrix with 387082 features) performed significantly worse (median R^2 = 0.047 95\% CI [0.044, 0.052]) than our stacking learning model."

This might also adversely affect the results presented: the stacked model, when using LASSO regularization, should produce winner-take-all kinds of behavior and indeed, in examining Figure 3, it looks as though weights on the different modality channels vary by quite a bit, even between samples that differ only by approximately 2% of the data (20

subjects / ~1000 subjects). This is worrisome, because it suggests that even with the large amount of data that was analyzed here, the stacked model is highly variable in its weighting of the different modalities, and thus that small shifts in the noise can drive weights extremely from one modality channel to another modality channel. This may be the reason for bimodal weight distributions in Figure 3. Granted, the data doesn't explicitly show this, so this is an inference, but one that could be refuted or validated.

Reply: We share these concerns raised by the reviewer. This in fact shows the necessity of testing the prediction performances against new partitions of the data, as we do here, in order not to report optimistic results that are only based on a very good partition of the data. In any case, our findings show that, in general, modalities that reliably contribute across cognitive domains like the local connectome and cortical surface area, and resting-state connectivity for fluid intelligence and volumetric factors for impulsivity are rather stable. Other modalities, like cortical thickness and subcortical volumes, whose predictive performance are in general smaller compared to the rest of modalities (see Figure 2), are likely more susceptible to noise and therefore can exhibit more unstable contributions. In general, such behaviours do not provide a significant contribution to stacking (median beta coefficient at a 95% confidence level), with maybe the only arguable case provided by cortical thickness for global cognitive score and cortical surface areas for Impulsivity (see Figure 3). Please, also note as mentioned at the beginning of this response, our second-level LASSO model now restricts to only nonnegative contributions, which may also render more stable solutions.

A question that arises is then: even if you stick to this stacked model, why is LASSO regularization even needed for the second level of analysis? It seems neccessary for the first level models, where the number of features is very large, sometimes even much larger than the number of subjects. But the second level model has only 5 features. It seems like the authors could fit an unregularized linear model to these features, which may prove more stable, and possibly also more accurate: essentially an optimally-weighted average of the predictions from the lower level. One kind of information that could help understand whether LASSO is required at this stage is a bit more insight into the optimal regularization parameter that was found at that level.

Reply: We agree with the reviewer that with only 5 features, in principle seeking for sparse solutions as those provided by LASSO models does not seem to be necessary. Indeed, other models like Ridge or even less conservative models like a plain ordinary least squares estimator, could have been employed. However, our motivation to include a L1 regularization term to our regression model at the stacked level was to directly account for the shared variance between single-channels. If multiple features are correlated, then the L1 regularization term picks the strongest feature as a representative of the group of correlated features which allows us to remove redundant channels, at least when taken in combination with others. Since this motivation was not clearly stated in the manuscript, we have added the following phrases to the beginning of the Results section:

“...The use of a LASSO model at this new learning level even with only five features (the number of single-channels) guaranteed that redundant modalities did not contribute to the final predictions...“

and to the Prediction Model sub-section in the Methods section:

"...The motivation to use a LASSO model with this small number of features (the number of single-channels) at this learning stage was to perform a feature selection as well, that automatically selected and weighted how much each channel contributed to the best final prediction..."

Relatedly, I found the description of the cross-validation procedure in the Methods to be a bit confusing. After looking through the code provided, I am convinced that this is done correctly, but the description in L555-L562 could raise doubts. In particular, I think that it would be good to clarify that the 70% mentioned on L557 are selected from within the 70% that were previously (L531) designated as the training set. If I understand correctly from my examination of the code, this is a form of nested cross-validation done only within the training set (within cell 49 of https://github.com/CoAxLab/multimodal-predict-cognition/blob/master/notebooks/05-predictions.ipynb). But the text around that part of the Methods is a bit ambiguous, and a reader might wonder whether the 70% in L557 are from a new partition of the entire dataset, which could risk leakage from the test set.

Reply: Yes, we are indeed doing a nested cross-validation even though we don't clearly state it in the text, so that no data leakage is taking place. We are aware of bad wording explaining this and we are sorry for the possible confusion created. Therefore, in order to be clearer about this crucial point and as we mentioned at the beginning of this response (in reply to reviewer 1) , we have added a new sub-section intended for details on the outer cross-validation procedure, changed the figure 1 which clearly shows now that the inner cross-validation, used for optimization, is performed only on the training data, and we have adjusted some parts of the "Prediction Models" sub-section.

Furthermore, one more suggestion. If the hierarchical nature of the stacked model is what the authors consider to be important in assessing the contributions of different modalities (i.e., the weights presented in Figure 3), there are still models that would provide this information, while still allowing the features at the first layer to interact with each other. For example, the authors might consider using a multi-layered perceptron, with L1 regularization (at the first, or both levels), to fit a hierarchical model. Again, one might expect better model performance, and more stability of second-level parameters across folds, with additional interpretability of relationships between the weights. I'll admit that this might be far out of scope for the current work, and could be discussed as potential future work. But at the very least, I think that it would improve the paper to add a comparison of the stacked architecture to simpler alternative model architectures (at least just a single-level PCA-LASSO or LASSO), which could demonstrate the utility of the stacking architecture in this setting.

Reply: As mentioned earlier, we have added a paragraph to the Discussion to better justify the use of our stacked learning approach. Moreover, this now includes a comparison with a single-level LASSO-PCR applied to the concatenated data across modalities for predicting global cognitive function score. As shown, the performance in this case (median R^2 = 0.047)

is worse than the performance achieved by our stacking approach. Yet, we'd like to highlight that this does not mean *per se* that stacking will always perform better.

About the suggested potential future work, we agree that algorithms like neural networks seem a compelling alternative where to improve our results. We had indeed thought about exploring these when designing the study, but we finally opted for using simple regressions models for their simplicity. In any case, we have added a few lines to the next-to-last paragraph in the Discussion section acknowledging the possible use of this kind of methods:

"...Alternatively, we speculate that a similar scenario as the one employed here could be accommodated using architectures of multi-layered neural networks. These models, however, require very large training sets, with tens of thousands of observations or more, which dwarfs most of the largest neuroimaging data sets currently available. Though, neural network approaches seem to be an appropriate future extension to our study as a stack of modality-wise neural networks, connected to a last hidden layer allowing for inter-channels connections, once appropriately large data sets become available."

Finally, a question regarding adjustments to the original variables. The authors chose to adjust the cognitive scores for age. I wonder whether similar adjustments would be applied to the brain data? In particular, whether cortical regional surface area should be normalized to overall cortical surface area.

Reply: As we have mentioned in the beginning of this response (First item in the list of major changes and second response to reviewer 1) and in our acknowledgements on the necessity for further accounting of confounds, we now also control for the intracranial volume. This in turn would control for associations between the independent and response data driven by overall cortical surface area, which is proportional to cortical surface area. Adjusting for confounders by residualizing on the response variable is sufficient to at least not get confounding predictions due to these third variables. Indeed, it's an usual approach adopted in predictive modelling studies, like for example in the ABCD Neurocognitive Prediction Challenge 2019 (https://sibis.sri.com/abcd-np-challenge/). See also the following references:

- A. Mihalik, M. Brudfors, M. Robu, F.S. Ferreira, H. Lin, A. Rau, T. Wu, S.B. Blumberg, B. Kanber, M. Tariq, M.D.M.E. Garcia, C. Zor, D.I. Nikitichev, J. Mourao-Miranda, N.P. Oxtoby, 2019. ABCD Neurocognitive Prediction Challenge 2019: predicting individual fluid intelligence scores from structural MRI using probabilistic segmentation and kernel ridge regression arXiv1905.10831 .
- Oxtoby, N. P., Ferreira, F. S., Mihalik, A., Wu, T., Brudfors, M., Lin, H., Rau, A., Blumberg, S. B., Robu, M., Zor, C., et al., 2019. ABCD Neurocognitive Prediction Challenge 2019: Predicting Individual Residual Fluid Intelligence Scores from Cortical Grey Matter Morphology. arXiv:1905.10834.

Minor comments:

In the Introduction, the authors cite the WU-Minn HCP, but the Methods point to the LONI DB and to the USC/MGH HCP. Based on the statistics of the data (e.g., number of participants), I do think that the WU-Minn HCP was used. Is that the case here?

Reply: Yes, as was also pointed by the other reviewers, we have mixed the information about both studies. We accordingly changed this information in the participants sub-section to just WU-MInn HCP, which is indeed the study used. We appreciate the reviewers for bringing up this mistake.

L57: Though this sentence points to the limitations of a lot of unimodal work, it's not always an implicit assumption that analyzing one type of data obviates other data. In other words, this is a bit strongly stated here.

Reply: We completely agree with the reviewer about this concern. We have thus relaxed this sentence by modifying the beginning of the paragraph where it was written as follows:

"Despite the success in applying predictive modeling approaches to the mapping of brain systems to individual differences in cognitive performance, previous work has largely focused on unimodal methods, which may not be sufficient to capture enough aspects of the brain due to the fact that different neuroimaging modalities reveal fundamentally distinct properties of underlying neural tissue...."

L353: "begs the question" does not mean that the question remains unanswered. Consider changing into "...this raises the question... " or some-such.

Reply: We agree with the reviewer and as such, we have modified this to the change suggested.

The sentence that starts on L382 is a bit unfortunately worded, because it's unclear which of the two (Pearson's or COD) is being referred to in the end of the sentence (Pearson's, presumably).

Reply: We have modified this wording, hoping that it is now clearer that we talk about the Pearson correlation coefficients as the non recommended metric for performance assessment in regression setups. The sentence now reads:

"Finally, we relied on the coefficient of determination, R^2, to assess the predictive power of the learned models. For regression tasks, this performance metric is recommended over the usual Pearson correlation coefficient, which overestimates the association between predicted and observed values [40, 41]."

I think that the authors used equation 5 to calculate the stacking bonus. So, it is not clear what we are supposed to make of equation 4. I also do not really understand how this relates to information and synergy among information transmitting channels. The text says "...joint system may convey more information than just the sum of its parts". I have two problems with this statement: (1) I don't think that R^2 is a good measure of information and (2) that's the average, not the sum. In other words: the connection to information theory is tenuous.

Reply: We apologize for the possible confusion caused. Our intention was not to connect this to information theory but to just give a kind of motivation for the introduction of our stacking bonus. In order to avoid any confusion, we have removed any mention to information theory. Now this paragraph reads as follows:

"Therefore, this quantity aims to estimate the difference in performance between the joint model and the average across its parts, which in our case correspond to the different brain measurement channels."

About equation 4, our intention was to just show how we ended up using equation 5, which looks more conservative than averaging across modalities. We chose to keep it here as some readers have found it helpful.

In looking at the GitHub repo, I noticed a commit "remove restrictive info". If this is information that should not be publicly available, the authors might want to rebase commits that include this file out of the history of their repository (e.g., https://github.com/CoAxLab/multimodal-predict-cognition/commit/2dab4b481c62314a09850de13ca92042a9cfa598)

Reply: We are really grateful to the reviewer for catching this. As suggested, we have rebased the commits to just showing the last commit, which did not include any restrictive info.

What are the units on the scales of the X axes in Figure S1? Are these axes somehow comparable to each other?

Reply: No, unfortunately as far as we are concerned they are not comparable in this case. The quantities shown in figure S1 correspond to the mean absolute error (MAE) and, as such, they are given in terms of the response variable units. For a direct comparison, it is better to resort to the coefficients of determination given in figure 2, which are normalized quantities.
